# Digital Smile Makeover: A Multidisciplinary Team Approach

**DOI:** 10.1055/s-0043-1764426

**Published:** 2023-05-02

**Authors:** Dalia Nourah

**Affiliations:** 1 Department of Basic and Clinical Oral Sciences, College of Dentistry, Umm Al- Qura University, Makkah, Saudi Arabia

**Keywords:** gummy smile, crown lengthening, digital technology, lip repositioning

## Abstract

Aesthetics is a fundamental part of contemporary dental practice. A pleasant smile depends on the gingival tissue architecture and dental characteristics. Excessive gingival display (gummy smile) is considered an unattractive smile and can affect a person's confidence. There are many etiological factors related to a gummy smile. Aesthetic rehabilitation of these cases often requires an interdisciplinary approach and close collaboration between dental specialties. This article describes an approach to excessive gingival display management caused by short teeth and hyperactive lips using a digital workflow for crown lengthening. A digital approach enables predictable planning and decreases the need for postsurgical modifications, thus shortening the treatment duration. Computer software is used for planning and 3D-printed guide for crown lengthening and implant placement. Two months later, lip repositioning was performed to reduce the hyperactive lip. After 4 months, prosthetic treatment and Botox injections were done to restore an aesthetic smile.

## Introduction

Nowadays, it has become common for patients to have high aesthetic demands, going beyond a simple desire for a smile makeover. 1 A “gummy smile,” commonly referred to as excessive gingival display (EGD), is a common aesthetic problem among dental patients. 2 3 The critical element in managing gummy smiles is identifying their etiology, which determines the treatment plan and outcomes. Patients may have one or more of the following etiologies: altered passive eruption (APE), where the gingival tissues had the incapacity to migrate apically, and the teeth appear short and square because the gingival tissues are coronal to the cementoenamel junction (CEJ). The other etiologies are related to short lips. The average lip length from the base of the nose to the inferior border of the upper lip is 20 to 22 and 22 to 24 mm in females and males, respectively. 4 The hyperactive upper lip is another etiology for a gummy smile. The lips should be examined while resting and smiling. Normal lip translation movement from rest is between 6 and 8 mm, while in the hyperactive lip, it can be up to 10 mm. 4 5 Skeletal etiologies include vertical maxillary excess (VME), defined as the excessive maxilla and dentoalveolar structure growth in an inferior direction. 6 The treatment modalities of a gummy smile are dependent on its etiology. The surgical management ranges from gingivectomy, modified lip repositioning, and orthognathic surgery. Less invasive treatment options include orthodontic therapy and botulin toxin injection. 7 In some cases, the gummy smile results from more than one factor; therefore, a combination of therapies is needed. 7 According to Andijani and Tatakis, gummy smile patients commonly have a combination of APE and hyperactive lip. 8 A crown lengthening surgery is performed to increase the clinical crown by removing a part of the gingival tissue, with or without bone sectioning. It is considered a common technique for restoring periodontal space in case of caries, fractures, and correcting aesthetic disorders. 9 For hyperactive lips, lip repositioning treatment (LRT) aims to narrow the vestibule and reduce the gingival display by restricting the muscle pull. It involves removing a strip of mucosa from the labial vestibule and making a partial-thickness flap between the mucogingival junction and the upper lip muscles. After this, the lip mucosa is sutured to the mucogingival line. 10 11 12 Botulinum toxin type A (BTX-A) has been introduced for the treatment of EGD, especially for those with hypermobility of the upper lip, as it blocks muscular contraction by inhibiting the release of acetylcholine in muscles. 13 Digital Smile Design (DSD) software helps in aesthetic zone diagnosis, planning, and treatment, particularly when full-arch rehabilitation is required. This technique also helps determine the exact location of the CEJ, allows precise measurement of the required reduction of tooth-supporting structures, and fulfils the aesthetic requirements. In addition, it facilitates communication between the dentist and the patient, which permits structuring the treatment plan to fit the patient's functional, aesthetic, and emotional needs. 14 This case report demonstrates a multidisciplinary treatment approach for EGD and smile rehabilitation utilizing digital technology in the diagnosis, planning, and treatment, as well as the treatment's durability for a 6-month follow-up.

## Case Report

A 38-year-old female patient presented to the dental clinic and expressed displeasure with her smile as she showed excessive gingiva display. The patient's medical history was noncontributory to the dental condition. Intraoral and extraoral photographs, as well as a video recording of maximum smile postures, were analyzed. Oral hygiene routine, habits, and previous dental treatments were also evaluated. Complete periodontal charting was recorded. Pocket depth ranged from 3 to 4 mm without evidence of clinical attachment loss. The required radiographs were obtained for the upper maxillary teeth. The bone level was at the level of the CEJ, and no bone loss was detected. Agreement from the patient was taken to publish the case and a written informed consent was obtained.

### Case Planning

**Fig. 1:**
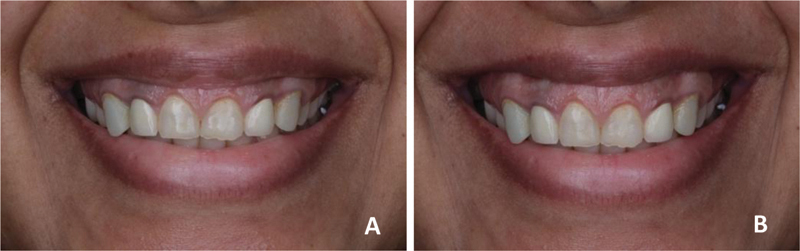
Patient smile. ( **A** ) At rest. ( **B** ) Full smile. Note the increased gingival display at the anterior teeth in the full smile. More than 4 mm of gingival was displayed at full smile. The anterior teeth were square shaped with a width-to-length ratio <80%, asymmetrical gingival margin, and chipped incisal edge and defective restoration.

Extraoral examination and facial analysis were done. A lateral cephalometric analysis confirmed the exclusion of the VME and dentoalveolar extrusion. The maxillary lip length was 21 mm, and the incisal display of the upper teeth at rest was 4 mm. The lateral and central incisors had a clinical crown height of 8 and 7mm, respectively. At the same time, the canine measured 8 mm. The probe-transparency test revealed a thick phenotype for the gingiva. The height of the alveolar bone crest for the upper arch was measured using a cone beam computed tomography (CBCT) scan (CS9300; Carestream Health Inc.). The patient's dense buccal cortex was also visible on the CBCT scan. Lip mobility showed a translation of 10 mm from rest to full smile. According to Coslet et al, the patient was diagnosed to have an EGD with short clinical crowns as a result of APE type 1 (gingival margin incisal to CEJ), subtype B (the alveolar bone at the level of the CEJ), and hyperactive lip ( Fig. 1 ). 6 9 In addition, the missing upper right first molar (tooth no. 16) was diagnosed.

The treatment plan included the following: (1) aesthetic crown lengthening procedure to restore the ideal length of the anterior teeth; (2) dental implant to replace the missing first molar, to be placed simultaneously with the crown lengthening procedure; (3) following healing, lip repositioning surgery to reduce the gingival display; and (4) final restorative work to improve anterior teeth shape and color and Botox injection.

### Planning and 3D Printing of Surgical Guide

**Fig. 2:**
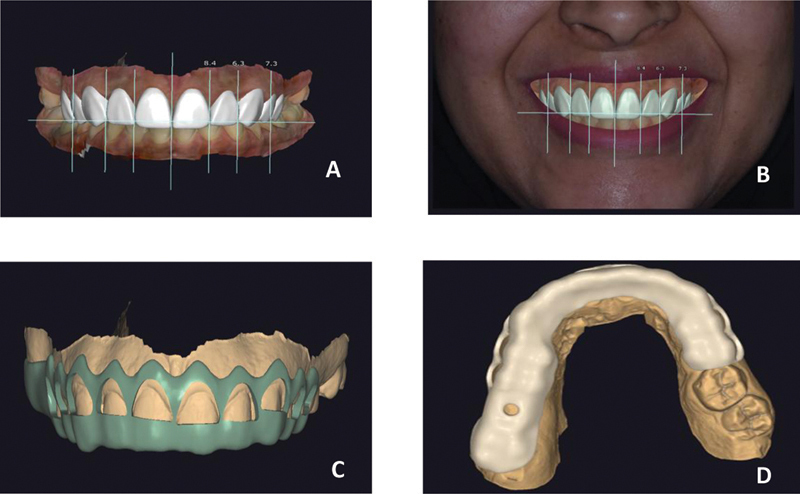
Smile design. ( **A** ) Two-dimensional clinical photography. ( **B** ) Three-dimensional digital model. ( **C** ) Designed guide on digital model. ( **D** ) Pilot drill design for implant placement to missing upper right first molar.

- Standardized photographs of the patient were taken using a DSLR camera with the following setting: mode—manual (M); aperture: f/15; shutter speed: 1/200; and autofocus (focus on the eye). The pictures consist of a front smile at rest, a full dynamic smile, a retracted front view with the teeth apart, and the patient's profile.
- An intraoral surface scan of the maxilla and an occlusal record in maximum intercuspal position were taken with an intraoral scanner (iTero Element 2) to obtain standard tessellation language (STL) files.
- CBCT (CS9300; Carestream Health Inc.) scan to obtain a digital imaging and communications in medicine (DICOM) file.
- Import the STL files from intraoral scanning, the RAW files from the photographs, and the DICOM file into a CAD-CAM software program (Exocad). Smile design and digital diagnostic wax-up were done ( Fig. 2A, B ).
- Virtual planning for the crown lengthening surgical guide that records the distance between the initial gingival margin and bone crest as a baseline measurement of the patient's biologic width. In addition, a pilot drill was done for implant placement at site no. 16 ( Fig. 2C, D ).
- A 3D printer (ProJet MJP 3600 Dental 3D systems) was used to print the surgical guide.

### Crown Lengthening Procedure

**Fig. 3:**
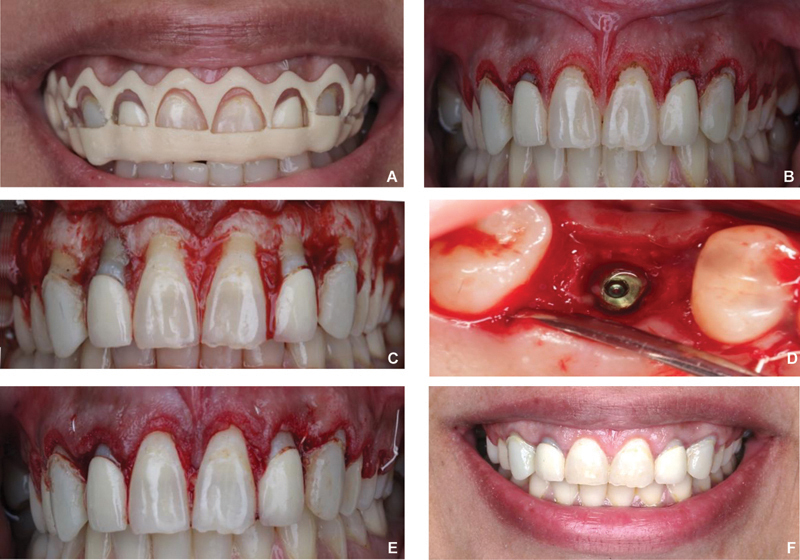
( **A** ) The crown-lengthening surgical guide positioned intraorally. The first level predicts the new gingival margin and determines the level for gingivectomy incision. The second level is a 3 mm apical to the first level, which determines the level of the alveolar crest (ostectomy). ( **B** ) Gingivectomy incision based on the predetermined level. A gingivoplasty was done to decrease the thickness of the tissue. ( **C** ) Full-thickness reflection, ostectomy, and osteoplasty were done. The new alveolar bone crest is 3 mm from the gingival margin. ( **D** ) Implant placement in site of the missing first molar (tooth no. 16). The implant is positioned to follow the new gingival margin after crown lengthening. ( **E** ) Flap reposition and suture. Immediate increase in the tooth length is noticed. ( **F** ) Eight weeks of follow-up. Decreased gingival display and harmonious gingival margin were noticed.

Local anesthesia was administered to the area from the upper-right second molar to the upper-left second molar (4% articaine with epinephrin 1:100,000). Surgical guide was tried and examined for fitting ( Fig. 3A ). A no. 15C (Bard parker blade, Medesy, Italy) blade was used for internal bevel incision (Gingivectomy) at the facial aspect of the right second premolar to the left first premolar. Care is taken to protect the interdental papillae ( Fig. 3B ). Once the desired gingival level was determined, the surgical guide was removed, and a full-thickness flap was reflected to visualize the alveolar bone. The surgical guide also helps determine the location of the new alveolar bone crest, which is 3 mm from the new gingival margin and therefore guides the bone removal (ostectomy) level. Ostectomy was done using a flat-tipped truncated cone drill (958 FG Surg End Cutting Carbide; BRASSELER USA) with smooth lateral surfaces and only an active tip allowing bone removal without touching the tooth structure. Osteoplasty and festooning of the interproximal hard tissue were done with a round diamond high-speed bur (801 FG diamond round, BRASSELER USA; Fig. 3C ). The surgical guide also allowed for accurate implant placement at the site of the upper-right first molar (BioHorizons, 4.2/10 mm, tapered implant; Fig. 3D ). Following irrigation, the flap was repositioned and sutured using 5–0 polypropylene (Ethicon, Inc., New Jersey, United States) using a combination of vertical mattress and interrupted suturing technique ( Fig. 3E ). Postoperative instructions regarding oral hygiene, chlorhexidine mouth wash, and pain control medication were given. Recall visits were scheduled for 2 and 8 weeks to evaluate soft-tissue healing, oral hygiene, and treatment outcomes. Favorable outcomes were reported, and the patient was satisfied with the results ( Fig. 3F ).

**Fig. 4:**
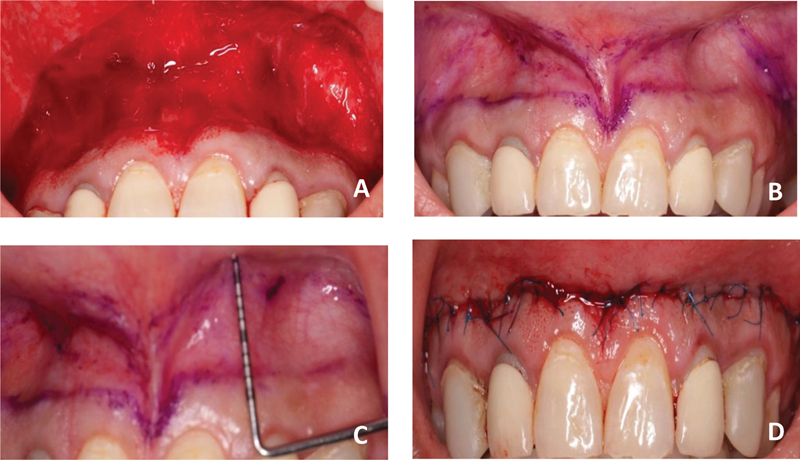
( **A,B** ) Incision area outlined using surgical skin marking. The first incision line is at the Mucogingival junction (MGJ) and the second line is 12 mm apical to the first one. This is determined based on the amount of lip repositioning required. ( **C** ) Superficial partial-thickness dissection to remove 1 mm of tissue thickness and performed through the frenum. Care was taken not to expose the bone. ( **D** ) Anchoring sutures at midline was first reestablished to ensure appropriate symmetry. The interrupted sutures were used to approximate the flap edges.

### Lip Repositioning Procedure

The patient was prepared for the second procedure 8 weeks after the crown lengthening surgery. Buccal infiltration was used to apply a local anesthetic solution to the vestibular region between the upper-right and the left first molar. A surgical skin marking pen (Securline) was used to outline the borders of the incision. The inferior border of the incision was placed at the mucogingival junction and was extended from the mesial aspect of the first premolars bilaterally ( Fig. 4A, B ). The superior line was 10 to 12 mm from the inferior incision line. The incision width is chosen based on how much of the last tooth is visible during a full, active smile in the horizontal direction. Partial-thickness incisions were made using a scalpel across the superior and inferior borders. Only 1 mm of tissue thickness is removed, with the connective tissue and muscle fibers remaining, and a strip of mucosa was peeled off as superficially as possible ( Fig. 4C ). Following that, suturing was performed utilizing a simple interrupted approach to help maintain appropriate symmetry, utilizing 5–0 polypropylene sutures (Ethicon, Inc.) for both stabilizing and anchoring sutures. The anchoring suture was first placed at the midline to aid in maintaining symmetry of the lip. Stabilizing sutures were utilized to bridge the gaps and hold the lip in its new location coronally ( Fig. 4D ).

The patient was given postoperative instructions with an emphasis on minimal lip movements for the first 3 to 4 days. Also, the patient was provided with pain control medication (ibuprofen 600 mg every 6 hours for 1 week). For 2 days, regular oral hygiene practices were abandoned. The patient was instructed to use 0.2% chlorhexidine gluconate mouthwash twice daily for 2 weeks.

### Follow-Up Appointments

**Fig. 5:**
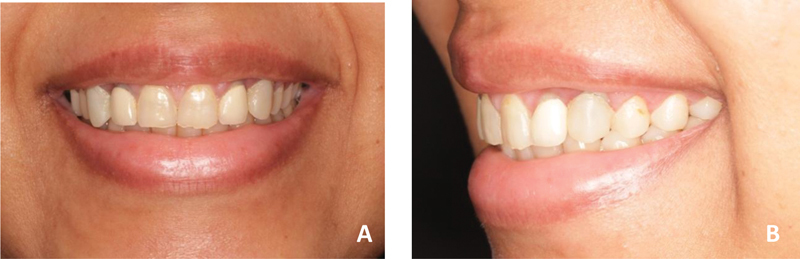
Two weeks of follow-up following lip repositioning. ( **A** ) Frontal smile view. ( **B** ) Side smile view. The gummy smile was covered.

**Fig. 6:**
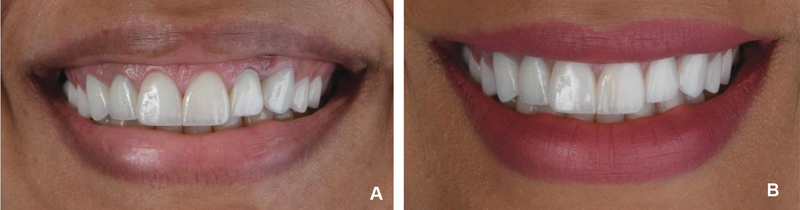
( **A** ) Four months post lip repositioning. Restorative treatment was done (crowns and veneers). Little relapse was noted: gingival tissue display 2 mm posteriorly and 1 mm anteriorly during the full dynamic smile. ( **B** ) Four units of botulinum toxin A (Allergan) was injected right and left of the area between the nose and the upper lip. The picture was taken 10 days after Botox injection.

Sutures were removed in the second week postoperatively. The surgical site had healed well at this point. The patient showed an average smile line. The patient was referred for removal of old restorative work and temporary restoration was placed. The patient was monitored for 4 months and then referred for completion of the final restorative work. There was a significant improvement in the patient's smile ( Fig. 5 ). A reassessment of the smile was done after completion of the restorative treatment. A 1- to 2-mm relapse was noticed ( Fig. 6A ). To manage that, 4 units of Botulinum toxin A (Botox, Allergan, United States) were injected right and left of the area between the nose and upper lip to reduce the appearance of gingiva at the posterior area based on the patient's request.

**Fig. 7:**
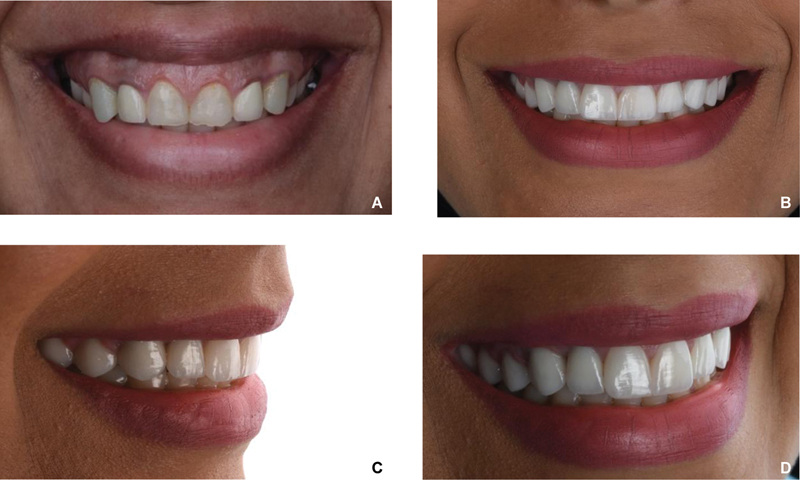
( **A** ) Before treatment. ( **B–D** ) Smile transformation after treatment.

Ten days following Botox injection, the patient's full dynamic smile showed significant improvement ( Fig. 6B ). In addition, before and after treatment pictures showed successful treatment outcomes in the patient's smile ( Fig. 7 ).

## Discussion

This case report describes the interdisciplinary approach for the management of EGD and restore an aesthetic smile using digital flow. In addition, different modalities to treat the advance EGD were used. The primary goal of aesthetic rehabilitation is creating a harmony between the teeth, gingiva, and lips. 15 16 Recent studies have revealed that the amount of gingival display during smiling affects the attractiveness of a smile. 17 Different etiologies for the gummy smile dedicate different treatment modalities. 5 In this case, the patient is diagnosed with a combination of APE and hyperactive lip. In addition, the patient needs full restorative rehabilitation to restore defective restorations and replace missing molar. Planning and management of such cases are challenging. Nowadays, the development of computer-aided design and 3D radiograph have improved and facilitated the treatment planning for complex interdisciplinary cases. 18 Software for smile analysis and 3D-printed surgical guides are invaluable tools to improve the accuracy and precision of clinical interventions and to increase predictability. 19 Crown lengthening is a surgical procedure commonly performed to increase the crown height and restore normal dentogingival relation. 20 A surgical guide is needed to confirm the margin of the new gingiva and bone. The conventional crown lengthening technique takes time while performing and modifying conventional wax-ups. It also has the added disadvantage of an unpredictable estimate of where the gingival margin should be. 14 In contrast, digital workflow allowed for more predictable crown lengthening planning and execution. 14 In this case, digital planning and 3D-printed surgical guides were used for crown lengthening and implant placement in site no. 16. It ensures limited changes in the hard and soft tissue and accurate results. However, digital workflow has some limitation as it needs well-trained laboratory technicians and long follow-up documentation.

Eight weeks after the crown lengthening procedure, a lip repositioning surgery was performed to manage the hyperactive lip and reduce the appearance of the gummy smile. There are different techniques of LRT. 10 In this case, an elliptical-shaped incision at the alveolar mucosa was done keeping the muscle fibers intact, and a 10- to 12-mm strip of mucosa was removed and the margins were sutured at the new level. After 2 months of healing (∼4 months after the crown lengthening surgery), restorative work was completed. A systematic review by Tawfik et al indicated that the average improvement in the gingival display from LRT was between 3 and 4 mm. 21 Reappearance and partial relapse were reported in the literature. For that, some authors suggested Botox injection as a combination treatment of gummy smile and long-term stability. 13 In this case, Botox injection was done to achieve the patient's requested smile. Several case reports on how to manage gummy smiles using digital technology have been published. 14 16 19 22 These cases present only one or two modalities for treating the gummy smile. On the other hand, our case report focused on the multidisciplinary management of the gummy smile using digital workflow as well as representing three different modalities to treat the gummy smile. Follow-up of 6 months with a pleasant result was documented. This indicates the importance of careful management of aesthetic cases and proper diagnosis as the keys for successful outcomes.

## Conclusion

The periodontist can plan and perform the crown lengthening procedure more precisely with the help of a 3D-printed guide, as it guides both bone and soft-tissue removal. The digital approach provides tools to obtain excellent treatment and facilitates communication between the dentist and the patient, particularly in challenging cases with high aesthetic demand and multiple etiologies such as the presented case. The current case report aims to describe the sequence of a multidisciplinary treatment of complex aesthetics using digital planning and different treatment modalities to achieve an aesthetic smile.
